# Remission of Suicidal Ideation in Emotionally Unstable Personality Disorder with Flupenthixol

**DOI:** 10.1155/2022/7097189

**Published:** 2022-01-13

**Authors:** Daniel J. Chivers, Mohammed Shaffiullah

**Affiliations:** ^1^University of Birmingham, UK; ^2^Birmingham and Solihull Mental Health Trust, UK

## Abstract

There are currently no licensed pharmacological treatments for Emotionally Unstable Personality Disorder. This case report describes a 50-year-old male who two years previously had been brought to the attention of psychiatric services following an overdose with intention to end his life. He was subsequently diagnosed with Emotionally Unstable Personality Disorder (EUPD) and, following further suicide attempts and trials of mainstream pharmacological treatments, responded to flupenthixol IM 20 mg fortnightly, experiencing complete remission from his suicidal ideation. Clinicians should be aware of EUPD presenting in later life and should consider the role of typical antipsychotics, including flupenthixol, in the treatment of suicidal ideation in patients with EUPD. Age-specific guidance on EUPD management would be of use to clinicians, especially in the management of older patients, as current guidance is based on findings within a narrow age group.

## 1. Introduction

The ICD-10 [1] describes Emotionally Unstable Personality Disorder as being characterised by the following:

- An enduring pattern
- Beginning by early adulthood
- Of unstable self-image and mood, volatile interpersonal relationships, self-mutilating behaviour, and impulsivity
- Including discrete episodes of loss of control of aggressive impulses
- With maladaptive patterns of behaviour.

The essential features of personality disorders are the impairment of interpersonal functioning, identity disturbance, and pathological personality traits. Many patients diagnosed with EUPD will enter remission and will no longer meet the criteria for diagnosis but may still receive treatment and be considered at high risk of decompensation [2].

There are some data that support the use of flupenthixol in the management of EUPD [3–6]. Specifically, flupenthixol and other typical antipsychotics have been demonstrated to reduce suicidality and irritability [7–9]. Atypical antipsychotics have also been demonstrated to be effective for treating acute agitation in patients with EUPD [10, 11]. Aripiprazole has also been demonstrated to reduce nonsuicidal self-injury (NSSI) in those without psychotic disorder and may therefore be a useful adjunct in the management of EUPD [12]. It is thought that the antipsychotic effects of flupenthixol are a result of D_2_ and/or 5-HT_2A_ antagonism [13].

The patient is a 50-year-old male who 2 years previously had been brought to the attention of services following an overdose. He was subsequently diagnosed with Emotionally Unstable Personality Disorder (EUPD) and, following further suicide attempts and trials of mainstream pharmacological treatments, responded to flupenthixol IM 20 mg fortnightly, experiencing complete remission from his suicidal ideation.

## 2. Case Presentation

The patient had a history of sexual abuse by a close family member between the ages of 6 and 19 and was bullied at school. He did not describe a childhood conduct disorder but reported that he struggled to make friends. He had childhood epilepsy, though is no longer on antiepileptics.

Following discharge from his first overdose, he reported several episodes of self-mutilation of the anus, penis, and urethra. He was initially treated with quetiapine and sertraline to manage his low mood, and promethazine, zolpidem, and lorazepam to manage insomnia and control impulsive behaviours. At this time, the patient declined Dialectical Behaviour Therapy, the primary treatment for EUPD. Despite medical intervention, the patient's mood remained low and labile, his behaviour remained impulsive, and his self-harm remained persistent. He presented to A&E three additional times following suicide attempts including hanging, a further overdose, and after jumping in front of a car.

The patient was started on IM flupenthixol 20 mg seven months after first presentation. The joint decision to trial flupenthixol was made with the patient as he was not responding to current treatment and based on previous anecdotal evidence for reducing self-harm and suicidal ideation in EUPD [3–6]. After treatment was commenced, his suicidal ideation subsided, and his mood stabilised. Subsequently, he also agreed to engage with Dialectical Behaviour Therapy. The patient is now stable on medication and has good insight into his improvement and premorbid condition. Though the use of antipsychotics is typically only recommended for short-term use in EUPD, it has been decided that he will remain on his current medications for the foreseeable future.

## 3. Discussion

Patients with EUPD typically begin treatment around the age of 18, though symptoms often begin earlier, with 60% of those with EUPD self-harming age 17 or younger [14]. No precursor syndrome has been identified specific to personality disorders, rather emotional dysregulation appears to be a spectrum that correlates with self-harm and suicidal thoughts [15]. Longitudinal studies have shown that remission in EUPD is slow. In those surviving over 15 years from diagnosis, 99% of patients experience >2 years of remission and after 27 years, 92% will no longer fit the criteria for personality disorder [14, 16].

There are currently no licensed medical treatments for EUPD [17]. A recent systematic review concluded that despite frequent use of medication in the management of EUPD, there is not yet convincing evidence of its efficacy [18]. In addition, there are no specific guidelines for the management of personality disorders in adults over the age of 50 [19]. The mainstay of treatment for EUPD is Dialectical Behaviour Therapy (DBT). DBT aims to help patients self-manage suicidal behaviour and has been shown to reduce self-directed violence [20]. In EUPD, medication plays the role of treating comorbid psychiatric conditions and managing troubling symptoms such as difficulty sleeping and anxiety.

Other pharmacological interventions, including lithium and clozapine, have been shown to reduce self-harm behaviour and improve mood stability in EUPD [21, 22]. However, lithium was considered inappropriate in this patient due to the risk of overdose. Both flupenthixol and clozapine were considered; however, as this patient suffered from childhood epilepsy, flupenthixol was favoured over clozapine as it has less potential to lower the seizure threshold [17]. A randomised controlled trial of the use of clozapine in EUPD is currently underway at the time of writing [23].

This patient presented at age 48. He described intrusive suicidal thoughts, fear of abandonment, and low mood since the destabilisation of his marriage. The patient had not experienced any symptoms of impulsivity, suicidal thoughts, or paranoid ideation prior to the trigger. We hypothesise that this patient's EUPD was latent; emerging due to decompensation of coping mechanisms and social support.

There is sparse literature on EUPD diagnosis and treatment in later life. Symptoms of EUPD vary through the life course, and cross-sectional studies appear to show that EUPD prevalence is lower in the older population. This can be partly accounted for by the increased risk of premature death in patients meeting the DSM criteria for EUPD [24]. Additionally, treatment studies have so far focused on those between the ages of 25 and 40. This highlights the need for research into and the production of age-specific guidance on diagnosis and management [5, 25, 26]. Screening and escalation of children with problematic childhoods may allow early intervention and prevention of EUPD decompensation in later life, as occurred in this case. However, this problem is multifaceted and difficult to achieve in practice.

In summary, clinicians should be aware of EUPD presenting in later life and should consider the role of typical antipsychotics, including flupenthixol, in the treatment of suicidal ideation, where other mood stabilisers may be unsuitable. Age-specific guidance on EUPD management would be of use to clinicians, especially in the management of older patients, as current guidance is based on findings within a narrow age group.

## Data Availability

The authors confirm that all relevant data are included in the article. Data were collected from Birmingham and Solihull Mental Health Trust.
